# SCAFE: a software suite for analysis of transcribed cis-regulatory elements in single cells

**DOI:** 10.1093/bioinformatics/btac644

**Published:** 2022-09-29

**Authors:** Jonathan Moody, Tsukasa Kouno, Jen-Chien Chang, Yoshinari Ando, Piero Carninci, Jay W Shin, Chung-Chau Hon

**Affiliations:** RIKEN Center for Integrative Medical Sciences, Yokohama City, Kanagawa 230-0045, Japan; RIKEN Center for Integrative Medical Sciences, Yokohama City, Kanagawa 230-0045, Japan; RIKEN Center for Integrative Medical Sciences, Yokohama City, Kanagawa 230-0045, Japan; RIKEN Center for Integrative Medical Sciences, Yokohama City, Kanagawa 230-0045, Japan; RIKEN Center for Integrative Medical Sciences, Yokohama City, Kanagawa 230-0045, Japan; Human Technopole, Milan 20157, Italy; RIKEN Center for Integrative Medical Sciences, Yokohama City, Kanagawa 230-0045, Japan; Genome Institute of Singapore, A*STAR Singapore, 60 Biopolis Street, Genome, #02-01, Singapore; RIKEN Center for Integrative Medical Sciences, Yokohama City, Kanagawa 230-0045, Japan

## Abstract

**Motivation:**

Cell type-specific activities of cis-regulatory elements (CRE) are central to understanding gene regulation and disease predisposition. Single-cell RNA 5′end sequencing (sc-end5-seq) captures the transcription start sites (TSS) which can be used as a proxy to measure the activity of transcribed CREs (tCREs). However, a substantial fraction of TSS identified from sc-end5-seq data may not be genuine due to various artifacts, hindering the use of sc-end5-seq for *de novo* discovery of tCREs.

**Results:**

We developed SCAFE—Single-Cell Analysis of Five-prime Ends—a software suite that processes sc-end5-seq data to *de novo* identify TSS clusters based on multiple logistic regression. It annotates tCREs based on the identified TSS clusters and generates a tCRE-by-cell count matrix for downstream analyses. The software suite consists of a set of flexible tools that could either be run independently or as pre-configured workflows.

**Availability and implementation:**

SCAFE is implemented in Perl and R. The source code and documentation are freely available for download under the MIT License from https://github.com/chung-lab/SCAFE. Docker images are available from https://hub.docker.com/r/cchon/scafe. The submitted software version and test data are archived at https://doi.org/10.5281/zenodo.7023163 and https://doi.org/10.5281/zenodo.7024060, respectively.

**Supplementary information:**

Supplementary data are available at *Bioinformatics* online.

## 1 Introduction

The expression of genes specifying cell identity is primarily controlled by the activities of their cognate cis-regulatory elements (CREs), mostly promoters (Forrest *et al.*, 2014) and enhancers (Andersson *et al.*, 2014). While gene expression can be quantified with single-cell RNA-sequencing methods (sc-RNA-seq), profiling of CREs primarily relies on single-cell Assay for Transposase Accessible Chromatin using sequencing (sc-ATAC-seq) (Buenrostro *et al.*, 2015). Alternatively, for a subset of CREs that are transcribed (i.e. tCREs), their transcription can be used as a proxy for their activity (Forrest *et al.*, 2014). Previously, we demonstrated the application of sc-end5-seq in the C1 platform (Fluidigm) for the detection of pre-annotated tCREs in single cells (Kouno *et al.*, 2019). However, *de novo* discovery of tCREs from sc-end5-seq data is challenging, due to excessive artifactual transcription start sites (TSS) arising from strand invasion (Tang *et al.*, 2013) and other sources (e.g. sequence biases) (Cvetesic *et al.*, 2018) during the template switching (TS) reactions (Adiconis *et al.*, 2018). This results in artifactual tCREs detected along the gene body known as ‘exon painting’ (Kawaji *et al.*, 2014). While a fraction of ‘exon painting’ reads could be attributed to cleavage and recapping (Affymetrix ENCODE Transcriptome Project and Cold Spring Harbor Laboratory ENCODE Transcriptome Project, 2009), their exact molecular origins remain elusive. Here, we have devised a multiple logistic regression classifier to effectively minimize artifactual TSS. It is implemented in a software suite, Single-Cell Analysis of Five-prime Ends (SCAFE), for *de novo* identification and annotation of tCREs from sc-end5-seq data.

## 2 Materials and methods

**Fig. 1.**
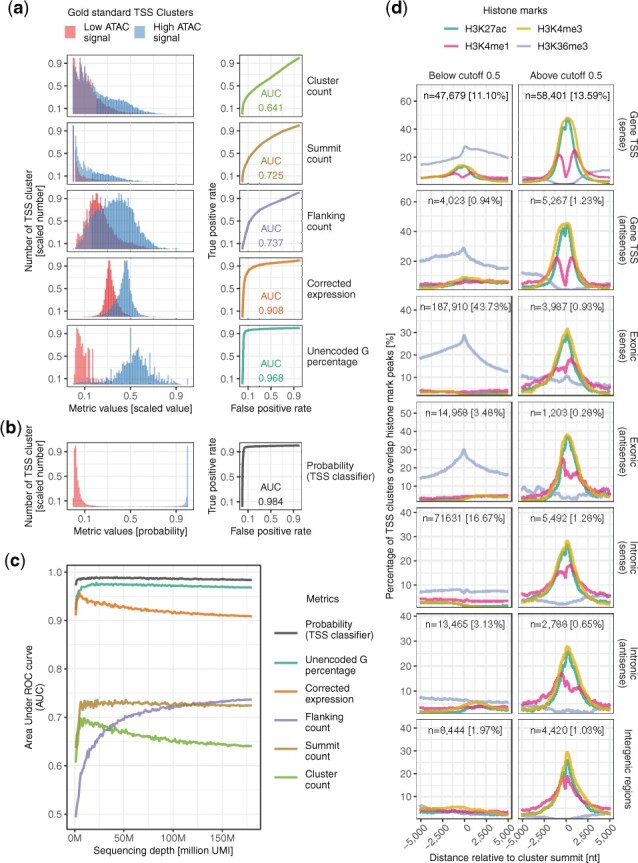
*De novo* identification of genuine TSS. (**a**) Distribution of TSS clusters properties (*left*) and their classification performances measured as AUC (*right*). (**b**) Distribution of probability (TSS classifier) (*left*) and its classification performance measured as AUC (*right*). (**c**) Performance of various metrics as a TSS classifier in (**a**) and (**b**) across various sequencing depths. (**d**) Histone marks at TSS clusters with a probability below (*left*) or above (*right*) 0.5 cutoff, at annotated gene TSS, exonic or intronic regions in sense or antisense orientations, or otherwise intergenic regions. *n*, number of TSS clusters; %, percentage of TSS clusters in all genomic locations regardless of probability thresholds

SCAFE consists of a set of command line tools written in Perl (Supplementary Fig. S1; Supplementary Table S1). SCAFE accepts read alignments *.bam files generated from *cellranger* (https://github.com/10XGenomics/cellranger) and read 1 should be sequenced for >35 genome-mappable nucleotides for confident identification of cDNA 5′ends. First, *scafe.tool.sc.bam_to_ctss* extracts the TS oligo/cDNA junction on read 1 and detects extra G mismatches (i.e. unencoded-G) at cDNA 5′end (Cumbie *et al.*, 2015) (Supplementary Note S1). This unencoded-G information will be later incorporated into a multiple logistic regression model to identify genuine TSS clusters. Then, *scafe.tool.cm.remove_strand_invader* removes the artifactual TSS due to strand invasion (Tang *et al.*, 2013) (Supplementary Note S2 and Supplementary Fig. S2a). Next, tool *scafe.tool.cm.cluster* defines TSS clusters by parametric clustering of cDNA 5′ends (i.e putative TSS) using *Paraclu* (Frith *et al.*, 2008) (Supplementary Note S3). Then *scafe.tool.cm.filter* extracts the properties of TSS clusters (Fig. 1a) and fits into a multiple logistic regression model (pre-trained or user-trained) to obtain probabilities for TSS classification (Fig. 1b). The multiple logistic regression model was trained to distinguish TSS clusters that are likely genuine (e.g. with high ATAC-seq signal, as true positives) and likely artifactual (e.g. with low ATAC-seq signal, as true negatives) (Fig. 1a; Supplementary Note S4). Users can supply their own epigenomic data for training (e.g. ATAC-seq signal), or use a model pre-trained with matched bulk-ATAC-seq and sc-end5-seq data on human iPSC. Next, *scafe.tool.cm.annotate* defines tCREs by merging closely located TSS clusters and annotates these tCREs as proximal or distal based on their distance to annotated gene TSS. It also defines hyperactive distal loci by stitching closely located distal tCREs with disproportionately high activities, analogous to super-enhancers (Chang *et al.*, 2019) (Supplementary Note S5). Finally, *scafe.tool.sc.count* counts the number of unique molecular identifiers (UMI) within each tCRE in single cells and generates a tCRE-by-cell UMI count matrix and *scafe.tool.cm.directionality* quantifies the strand biases of their expression (Supplementary Note S6). Workflows are available for various user scenarios, e.g. aggregating multiple libraries, and other tools available for processing bulk 5′end RNA-sequencing data (Shiraki *et al.*, 2003).

## 3 Results

We have used SCAFE to identify tCREs from sc-end5-seq data on human iPSCs and benchmarked with matched bulk—ATAC-seq and bulk-CAGE data (Supplementary Note S7; genome browser view available, see ‘Data availability’). About 3% of reads were identified as strand invasion artifacts and removed (Supplementary Fig. S2b). TSS clusters were defined (*n* = 429 668). We observed a substantially higher proportion of TSS clusters along the gene body in sc-end5-seq methods than bulk-CAGE (Supplementary Fig. S3a), consistent with the fact that ‘exon painting’ is more prevalent in TS-based methods (Cumbie *et al.*, 2015). We benchmarked the properties of TSS clusters for the classification of genuine and artifactual TSS clusters (Fig. 1a). The UMI counts within the TSS cluster (cluster count) performed the worst [area under receiver operating characteristic (ROC) curve (AUC) = 0.641], and its performance decreased with sequencing depth (Fig. 1a and c). Two other common metrics, UMI count at TSS summit (summit count, AUC = 0.725) and within ±75nt flanking its summit (flanking count, AUC = 0.737) performed only marginally better than the cluster count (Fig. 1a), suggesting these commonly used metrics are at best mediocre classifiers for TSS. As ‘exon painting’ artifacts are positively correlated with transcript abundance, making count-based thresholds poor performers, we examined other metrics that are independent of transcript abundance, including UMI counts corrected for background expression (corrected expression) and percentage of reads with 5′mismatched G (Cumbie *et al.*, 2015) (unencoded-G percentage) (Supplementary Note S4). Notably, both metrics performed well across sequencing depths with AUC >0.9 (Fig. 1c).

We found the combination of flanking count, unencoded-G percentage and corrected expression achieved the best performance. Therefore, these three predictors were used to devise a combined TSS classifier using multiple logistic regression (Fig. 1b), which achieved AUC >0.98 across sequencing depths (Fig. 1c). Its accuracy is high for TSS clusters at various genomic regions across a wide range of cutoffs (Supplementary Fig. S4a), which is well-validated by chromatin accessibility, promoter motifs, CpG island, sequence conservation (Supplementary Fig. S4b–f) and histone marks (Fig. 1d). At the default cutoff (probability = 0.5), ∼98% of sense exonic TSS clusters were removed (Supplementary Fig. S4a). These removed TSS clusters are void of marks for active CREs but overlap marks for transcription elongation, suggesting our combined TSS classifier effectively removed ‘exon painting’ artifacts (Fig. 1d). In addition, the TSS clusters located at gene TSS are marked with a bimodal H3K4me1 pattern which indicates active promoters, in contrast to the others that are marked with relatively unimodal H3K4me1 pattern which indicates active enhancers (Cheng *et al.*, 2014) (Fig. 1d). Finally, tCREs (*n* = 34 684) were defined as either proximal (*n* = 24 808) or distal (*n* = 9878) based on their distance to annotated gene TSS (Supplementary Fig. S3b). The genome-wide distribution of tCREs defined by sc-end5-seq and bulk-CAGE data are similar (Supplementary Fig. S3c). Considering the excessive exonic TSS cluster in sc-end5-seq before filtering (Supplementary Fig. S4a), our combined TSS classifier effectively minimize these ‘exon painting’ artifacts, which cannot be easily distinguished from genuine TSS clusters solely using count-based metrics (Fig. 1a). Our combined TSS classifier thus provides an integrated metric that is mostly independent of RNA expression levels and robustly distinguishes genuine TSS from artifacts.

## Supplementary Material

btac644_Supplementary_Data

## Data Availability

The sc-end5-seq, bulk-ATAC-seq and bulk-CAGE data of human iPSC used for benchmarking were deposited on The European Nucleotide Archive (ENA) under the accessions ERR5858616, ERR5856252 and ERR5774727 respectiviely. These data can be interactively explored on ZENBU genome browser at https://fantom.gsc.riken.jp/zenbu/gLyphs/#config=tCRE.browser.benchmark_tssCluster.iPSC_end5_dT.
