# Supplementary material for: SCAFE: a software suite for analysis of transcribed cis-regulatory elements in single cells
Source: Bioinformatics. 2022 Sep 29;38(22):5126–8. doi: 10.1093/bioinformatics/btac644 (PMC9665856; doi:10.1093/bioinformatics/btac644)
Supplement: btac644_Supplementary_Data [file btac644_supplementary_data.pdf]

SCAFE: a software suite for analysis of transcribed *cis*-regulatory elements in single cells

J. Moody, T. Kouno, J.C. Chang, Y. Ando, P. Carninci, J.W. Shin and C.C. Hon

Supplementary Information

Table of contents

Supplementary Table 1. A list of scripts in SCAFE.

Supplementary Fig. 1. Overview of SCAFE.

Supplementary Fig. 2. Detection of strand invasion artifacts.

Supplementary Fig. 3. Genome distributions of read/UMI and tCRE.

Supplementary Fig. 4. Properties of logistic model probability cutoffs.

Supplementary Note 1. Identification of junction between TS oligo and cDNA.

Supplementary Note 2. Identification of strand invader.

Supplementary Note 3. Clustering of cDNA 5'ends and extraction of TSS cluster properties.

Supplementary Note 4. Building of a TSS classifier with multiple logistic regression.

Supplementary Note 5. Annotation of tCREs.

Supplementary Note 6. Directionality of tCREs.

Supplementary Note 7. Datasets used in this study.

Supplementary Table

Script	Type	Context
scafe.workflow.sc.subsample	workflow	single-cell mode, subsample ctss
scafe.workflow.sc.solo	workflow	single-cell mode, process a single sample
scafe.workflow.cm.aggregate	workflow	commond mode, aggregate multiple samples
scafe.workflow.bk.subsample	workflow	bulk mode, subsample ctss
scafe.workflow.bk.solo	workflow	bulk mode, process a single sample
scafe.tool.sc.subsample_ctss	tool	single-cell mode, subsample ctss
scafe.tool.sc.count	tool	single-cell mode, count of UMI within tCRE
scafe.tool.sc.bam_to_ctss	tool	single-cell mode, convert bam to ctss
scafe.tool.cm.remove_strand_invader	tool	common mode, remove strand invader artefact
scafe.tool.cm.prep_genome	tool	common mode, prepare custom reference genome
scafe.tool.cm.filter	tool	common mode, filter for genuine TSS clusters
scafe.tool.cm.directionality	tool	common mode, calculate directionality of tCREs
scafe.tool.cm.ctss_to_bigwig	tool	common mode, convert ctss to bigwig
scafe.tool.cm.cluster	tool	common mode, cluster ctss
scafe.tool.cm.annotate	tool	common mode, define and annotate tCRE
scafe.tool.cm.aggregate	tool	common mode, aggregate ctss of multiple samples
scafe.tool.bk.subsample_ctss	tool	bulk mode, subsample ctss
scafe.tool.bk.count	tool	bulk mode, count ctss within tCREs
scafe.tool.bk.bam_to_ctss	tool	bulk mode, convert bam to ctss bed
scafe.download.resources.genome	others	download reference genome to resources directory
scafe.download.demo.input	others	download demo input data for testing
scafe.demo.test.run	others	run demo data for testing
scafe.check.dependencies	others	check dependencies

Supplementary Table 1. A list of scripts in SCAFE.

Supplementary Figures

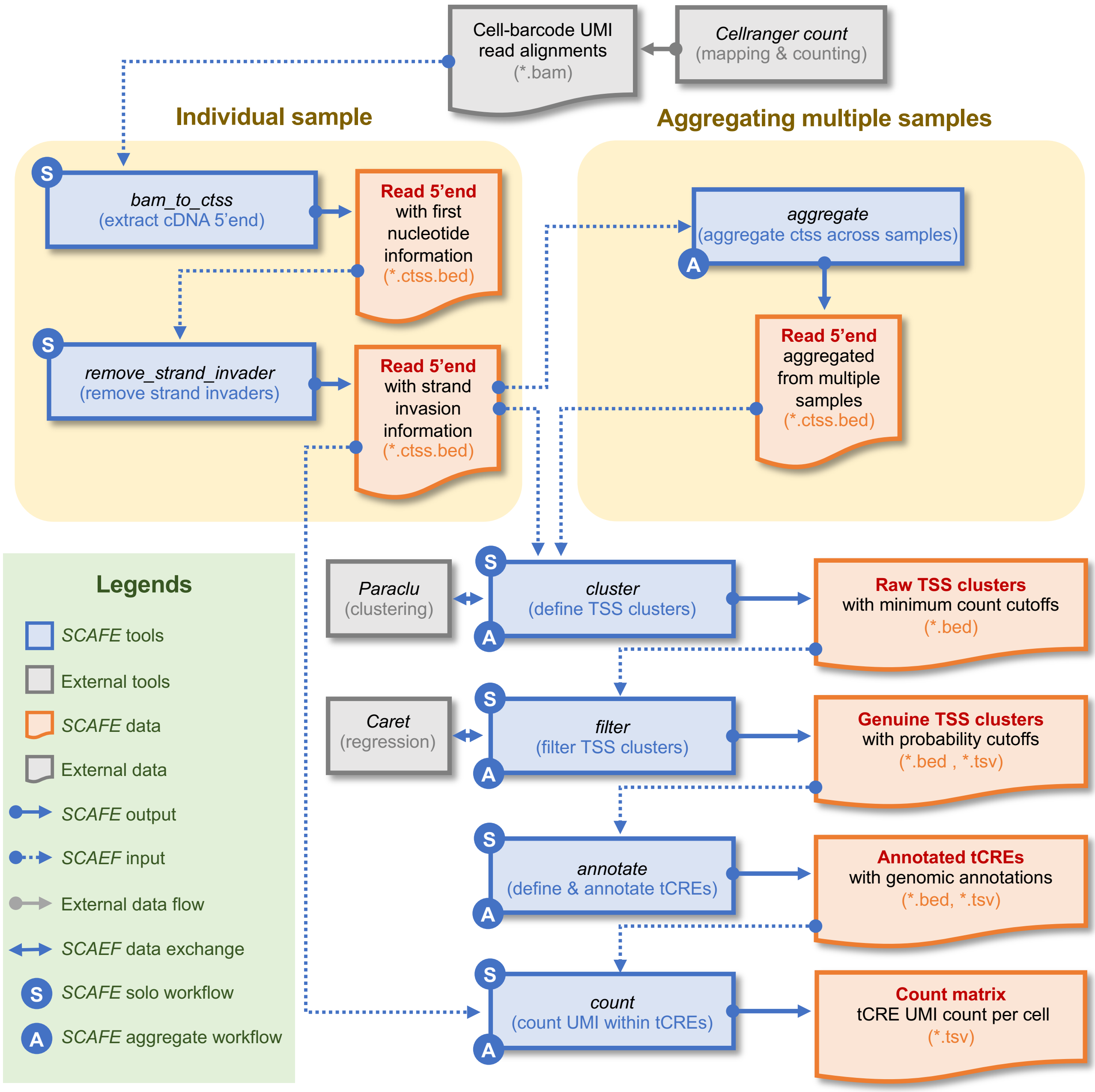

Supplementary Fig. 1. Overview of *SCAFE*. *SCAFE* consists of a set of *perl* programs for processing of sc-5end-seq data. Major tools are listed here, for a full list please visit https://github.com/chung-lab/SCAFE. *SCAFE* accepts read alignment in. *bam* format from *cellranger* (https://github.com/10XGenomics/cellranger). Tool *bam_to_ctss* extracts the 5′end of cDNA, taking the 5′ unencoded-Gs into account. Tool *remove_strand_invader* removes cDNA 5′ends that are strand invasion artifacts by aligning the TS oligo sequence to the immediate upstream sequence of the cDNA 5′end. Tool *cluster* performs clustering of cDNA 5′ends (i.e. putative TSS). Tool *filter* extracts the properties of TSS clusters and performs multiple logistic regression to distinguish genuine TSS clusters from artifacts. Tool *annotate* defines tCREs by merging closely located TSS clusters and annotates tCREs based on their distance to annotated gene TSS. Tool *count* counts the number of UMI within each tCRE in single cells and generates a tCRE-Cell UMI count matrix. *SCAFE* tools were also implemented workflows for processing of individual samples (*solo* workflow) or aggregating of multiple samples (*aggregate* workflow).

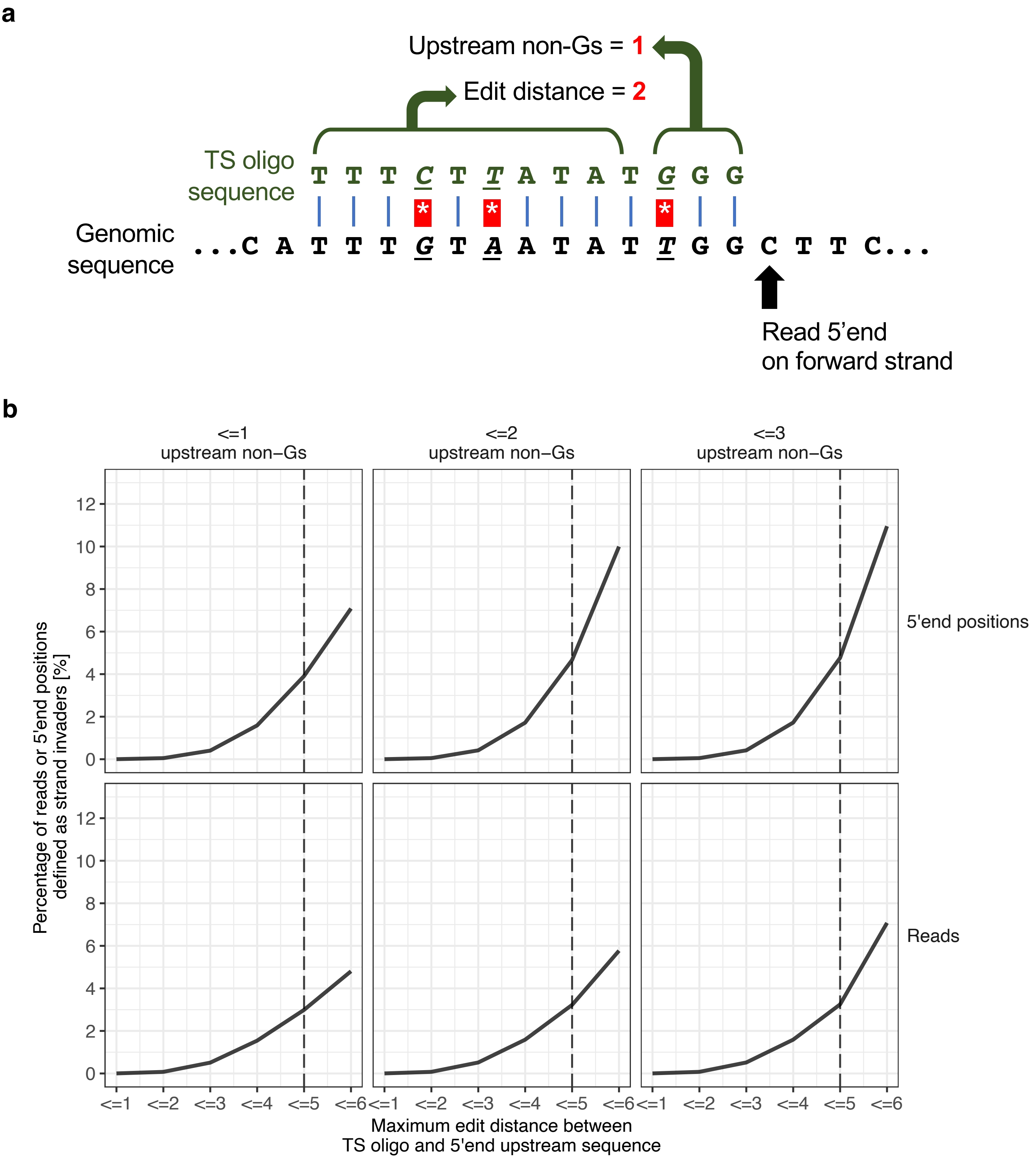

Supplementary Fig. 2. Detection of strand invasion artifacts. a, Rationale of strand invasion detection. The immediate upstream sequence of the read 5′end was aligned with the TS oligo sequence. Number of upstream non-Gs was calculated from the first 3nt of the immediate upstream sequence. Edit distance was calculated from the last 10 nt of the alignment. The shown example has 2 edit distances and 1 upstream non-Gs. **b**, Extent of strand invasion artifacts in sc-end5-seq. Maximum edit distance of 5 (*vertical dotted line*) and 2 upstream non-Gs (*middle column*) is chosen as the threshold to define strand invasion artifacts. At this threshold, ~3% of reads and ~4.8% of 5'end positions were regarded as strand invasion artifacts.

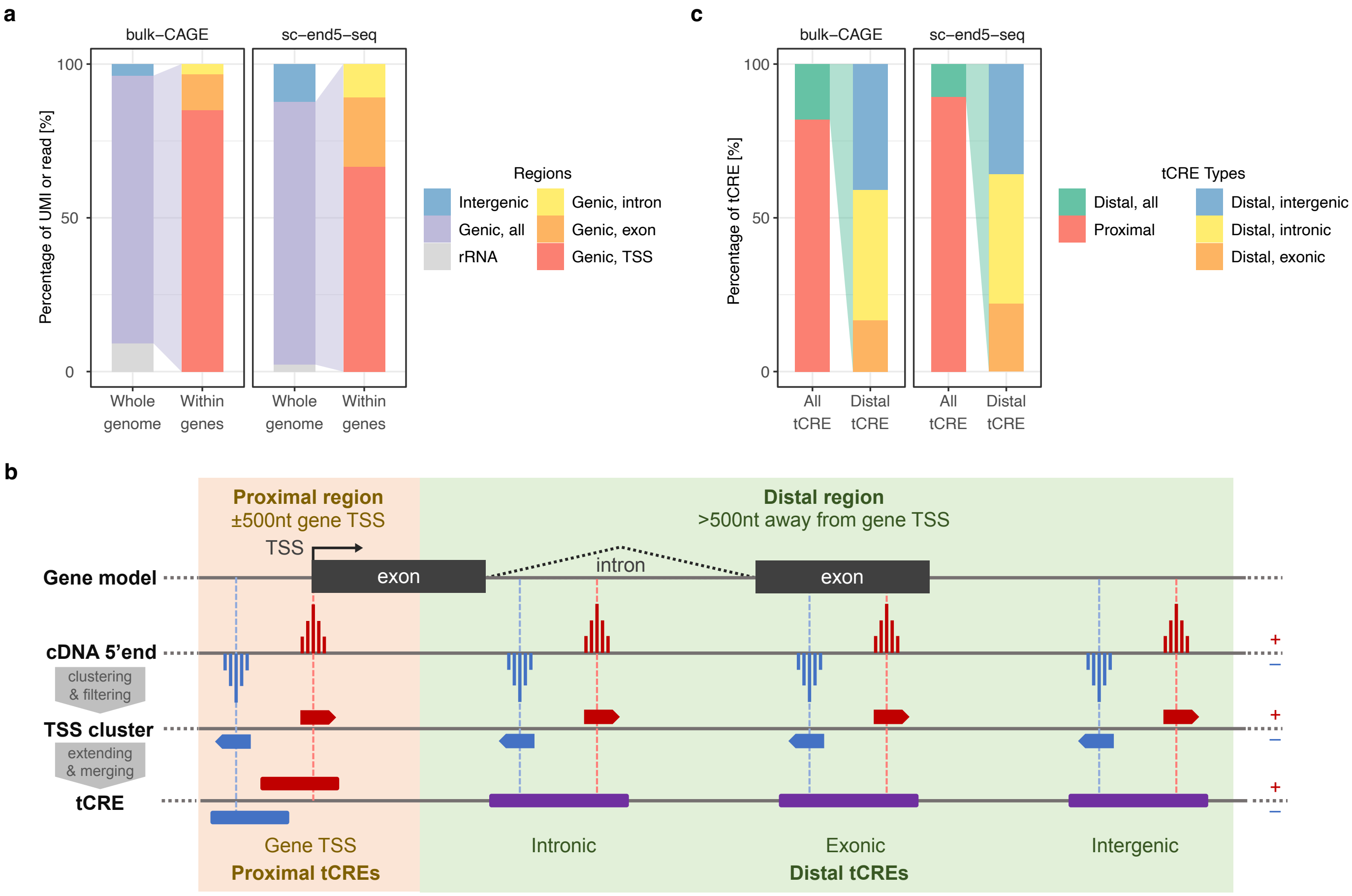

Supplementary Fig. 3. Genome distributions of read/UMI and tCRE. **a,** Proportion of read (bulk-CAGE) or UMI (sc-end5-seq) aligned to various genomic regions. **b,** Schematic defining of tCRE by merging closely located TSS clusters. Distance to gene TSS was used as the criteria to define proximal or distal TSS clusters and tCREs. Proximal and distal TSS clusters were merged in stranded and strandless manner, respectively. Distal tCREs are further classified as intronic, exonic, or otherwise intergenic. **c,** Percentage of tCRE from bulk-CAGE and sc-end5-seq assigned as proximal or distal, and within distal as exonic, intronic or intergenic.

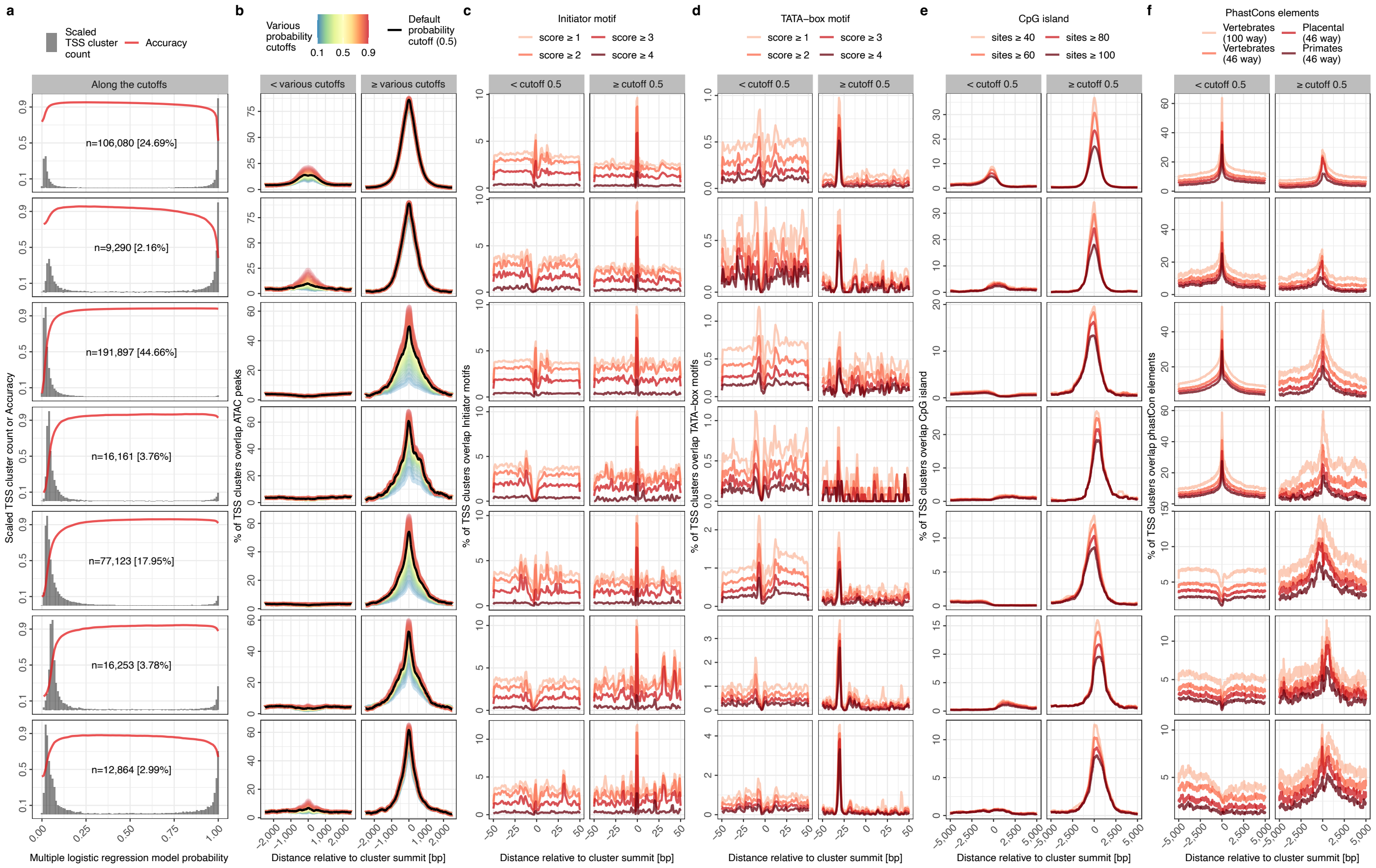

Supplementary Fig. 4. Properties of logistic model probability cutoffs for identification of genuine TSS clusters. **a,** Proportion of TSS clusters and accuracy along logistic model probability cutoffs. "n" and "%" refers to the number and percentage of TSS clusters in the category. **b,** Chromatin accessibility around the summit of TSS clusters along multiple logistic regression model probability thresholds. **c,d,e,f,** Distribution of Initiator motif, TATA-box motif, CpG island and PhastCons (Siepel *et al.*, 2005) elements, respectively, around the summit of TSS clusters below and above multiple logistic regression model probability 0.5. Initiator motif and TATA-box motif were predicted on hg19 using *HOMER* (Heinz *et al.*, 2010) (http://homer.ucsd.edu/homer/motif/). CpG island and PhastCons elements were downloaded from UCSC table browser (https://genome.ucsc.edu/). "Score" in *c* and *d* refers to the score of motif prediction from *HOMER*. "Sites" in *e* refers to the number of CG dinucleotides. In *f*, 100 way and 46 way refer to multiple genome alignments of 100 and 46 species respectively. Vertebrates, Placental and Primates refer to the scope of species used to define PhastCons elements. Initiator motif and TATA-box motif are, as expected, enriched at ~ 0nt and ~ –30nt, respectively, of the TSS cluster above probability cutoff 0.5. The enrichment of PhastCons elements at the center of the "Gene TSS" and "Exonic" TSS clusters below probability cutoff 0.5 can be attributed to their overlap with exon regions, which are relatively more conserved than intronic and intergenic regions.

Supplementary Notes

Supplementary Note 1. Identification of junction between TS oligo and cDNA

Previous studies suggest most reads derived from capped RNAs begin with an unencoded G, which can be used to distinguish genuine TSS from artifacts (Kawaji *et al.*, 2014; Cumbie *et al.*, 2015). To precisely calculate the number of unencoded-G for each mapped read, we first identify the junction between TS oligo and cDNA sequence and then examine the cDNA 5′end. The 5′end of cDNA was defined as the first nucleotide immediately following the last nucleotide of the TS oligo sequence. The first 3nt of cDNA sequence was compared to the genomic sequence at their corresponding aligned position, and the number of Gs that are mismatched was defined as the number of unencoded-G for the examined read. We provide 3 modes for determining this junction 1) "match": search for TS oligo sequence on the read, identify the TSO/cDNA junction as 5'end of the read. This works only when the error rate of the TS oligo region on the read is low, otherwise a considerable number of reads will be invalid. 2) "trim": assuming the 1st N bases of the reads are TS oligo, without checking the actual sequence. N is determined by the length of TS oligo. 3) "skip": assuming the TS oligo was not sequenced, the 1st base of the read will be treated as the 1st base after the TS oligo. A 4th mode "auto" will automatically determine the best mode, based on the observed error rate of the TS oligo and the frequency of 5'end soft-clipped bases by the aligner. If soft-clipped bases are close to the length of TS oligo, mode 1 or 2 will be chosen, depending on the observed error rate of the TS oligo (error rate ≤ 0.1, mode 1 will be chosen or mode 2 otherwise). If soft-clipped base is close to zero, mode 3 will be chosen.

Supplementary Note 2. Identification of strand invader

Strand invasion artifacts, i.e., strand invaders, can be identified based on complementarity of genomic sequence upstream of the mapped reads to TS oligo sequence, according to a study (Tang *et al.*, 2013), see also 10x Genomics technical note (https://support.10xgenomics.com/permalink/3ItKYUsoESnDpnFNnfgvNT). Briefly, we extracted a 13nt genomic sequence immediately upstream of the 5′end of cDNA, then globally aligned to the TS oligo sequence (TTTCTTATATGGG) and calculated the edit distance. A read is considered as an artifact of strand invasion when 1) the edit distance ≤5 and two of the three nucleotides immediately upstream were guanosines (Supplementary Fig.2a), based on the previously proposed thresholds (Tang *et al.*, 2013).

Supplementary Note 3. Clustering of cDNA 5'ends and extraction of TSS cluster properties

The 5′end of cDNA (i.e. putative TSS) were extracted as described above, deduplicated as UMI, piled-up and clustered with *Paraclu* (Frith *et al.*, 2008) using default parameters. Only the TSS clusters with total UMI ≥5 and summit UMI ≥3 were retained. The following properties were extracted for each TSS cluster: 1) cluster count, 2) summit count, 3) flank count, 4) corrected expression and 5) unencoded-G percentage. Cluster, summit and flank count refers to UMI counts within the cluster, at its summit, and within a region flanking its summit (±75nt). Corrected expression refers to an expression value relative to its local background, based on the assumption that the level of exon painting artifacts are positively correlated with the transcript abundance. Specifically, if the summit of a TSS cluster is located within genic regions, it will be assigned to either exon or intron, in either sense or antisense strand of the corresponding gene, or otherwise assigned to intergenic, as its local background. All annotated TSS regions (±250nt) were masked from these local backgrounds. The density of UMI per nucleotide within each local background is calculated (i.e., local background density). The corrected expression of a TSS cluster is calculated as the ratio of the density of UMI within the region flanking its summit (±75nt) to the density of its local background. Unencoded-G percentage refers to the percentage of UMI within the cluster that has ≥1 unencoded-G.

Supplementary Note 4. Building of a TSS classifier with multiple logistic regression

To combine multiple properties of a TSS cluster into a single classifier, we used multiple logistic regression implemented in the *caret (Kuhn, 2008)* R package. For training of this classifier, we defined a set of "gold standard" TSS clusters based on their bulk-ATAC-seq signal (as mean –log(P) within the TSS cluster). Specifically, the top and bottom 5% of TSS clusters, ranked by their bulk-ATAC-seq signal, were defined as positive and negative gold standards, and used for training of the multiple logistic regression models at 5-fold cross-validation. The resulting probability was used as the TSS classifier. The performance of this TSS classifier, and its constituent metrics, is measured as AUC, using the top and bottom 10% of TSS clusters as positive and negative gold standards for testing. The cutoff of probability at 0.5 is defined as the default threshold. All the TSS clusters in this study are filtered with this default cutoff.

Supplementary Note 5. Annotation of tCREs

tCREs are defined by merging closely located TSS clusters. Briefly, TSS clusters located within ±500nt of annotated gene TSS were classified as proximal, or as distal otherwise. All TSS clusters were then extended 400nt upstream and 100nt downstream. These extended ranges were merged using *bedtools (Quinlan and Hall, 2010)*, in a strand-specific manner for proximal TSS clusters and non-strand-specific manner for distal TSS clusters, as proximal and distal tCRE respectively. Distal tCRE were then assigned to either exonic, intronic or intergenic, in this order. Distal hyperactive loci analogous to super-enhancers (Whyte *et al.*, 2013) were defined by stitching together distal tCRE within a user specified distance, and ranking these in ascending order of UMI count, a tangent of this line is used to define the cutoff for defining a distal hyperactive locus. Potential alternative promoters beyond reference transcript 5'ends were defined as intronic or exonic tCREs containing 10% or more of the total UMI assigned to a gene (proximal, intronic and exonic tCREs).

Supplementary Note 6. Directionality of tCREs

Directionality of a tCRE is a measure of the bias of signal between the two strands. As a note, transcribed enhancers are generally bidirectionally transcribed. SCAFE first identifies the summits of signal on both strands with each tCRE, then count the read/UMI counts downstream of these summits and calculates the directionality as the following:

directionality = | plus stand signal - minus stand signal | / (plus stand signal + minus stand signal)

If the minus strand signal summit is upstream of the plus strand signal summit, its orientation is defined as divergent, or otherwise convergent. A negative sign will be added to the directionality value if its orientation is convergent. By definition, a value of 1 indicates the tCRE is perfectly bidirectional (i.e. same signal strength on both strands) in a divergent orientation. A value of –1 indicates the tCRE is perfectly bidirectional in a convergent orientation. A value of 0 indicates the tCRE is purely unidirectional (i.e. signal found on only one strand).

Supplementary Note 7. Data used in this study

Preparing Human iPSC samples. iPSC (Fort *et al.*, 2014) were cultured in StemFit medium (Reprocell) under feeder-free conditions at 37°C in a 5% CO_2 incubator. The cells were plated on dishes pre-coated with iMatrix-511 (Nippi). Rock inhibitor Y-27632 (FUJIFILM Wako) was added to the cells at a final concentration of 10µM during the first day of culturing. StemFit medium is refreshed daily until harvesting. The cells were detached and dissociated by incubating with TrypLE Select (Thermo Fisher Scientific) followed by scrapping in StemFit medium. The cells were collected by centrifuge and washed twice with 0.04% BSA in PBS.

Preparing sc-RNA-seq libraries for iPSC. Freshly prepared iPSCs were loaded onto the Chromium Controller (10x Genomics) on different days. Cell number and viability were measured by Countess II Automated Cell Counter (Thermo

Fisher Scientific). Final cell density before loading was adjusted to 1.0×10^6 cells/ml with >95% viability, targeting ~5,000 cells. Briefly, single cell suspensions were mixed with Single Cell Master Mix containing oligo(dT) primer (AAGCAGTGGTATCAACGCAGAGTAC–T(30)–VN) and loaded together with 5′gel beads and partitioning oil into a Chip A Single Cell according to the manufacturer's instructions. RNAs within single cells were uniquely barcoded and reverse transcribed within droplets. We used Veriti Thermal Cycler (Applied Biosystems) for RT reaction at 53ºC for 45 minutes. Then, cDNAs from each method were amplified using cDNA primer mix from the kit, with 12 PCR cycles, followed by the standard steps according to manufacturer's instructions. Libraries were barcoded by different indexes from i7 sample index plate (10x Genomics). The libraries were examined in Bioanalyzer (Agilent) for size profiles and quantified by KAPA Library Quantification Kits (Kapa Biosystems). All libraries were sequenced on HiSeq 2500 (Illumina) as 75 bp paired-end reads.

Preparing bulk-CAGE and bulk-ATAC-seq libraries. Bulk-CAGE libraries were generated by the nAnT-iCAGE (Murata *et al.*, 2014) method as previously described and sequenced on HiSeq 2500 (Illumina) as 50bp single-end reads. Bulk-ATAC-seq was performed as previously described (Buenrostro *et al.*, 2015) with slight modifications. Briefly, 25,000 cells were used for library preparation. Due to the more resistant membrane properties of DMFB cells, 0.25% IGEPAL CA-630 (Sigma-Aldrich) were used for cell lysis. Transposase reaction was carried out as described followed by 10 to 12 cycles of PCR amplification. Amplified DNA fragments were purified with MinElute PCR Purification Kit (QIAGEN) and size-selected with AMPure XP (Beckman Coulter). All libraries were examined in Bioanalyzer (Agilent) and quantified by KAPA Library Quantification Kits (Kapa Biosystems). Bulk-ATAC-seq libraries were sequenced on HiSeq 2500 (Illumina) as 50bp paired-end reads.

Processing sc-end5-seq data. Reads were aligned to hg19 with *cellranger v3.1.0* (10x Genomics).

Processing bulk-CAGE data. Reads were aligned to hg19 with *hisat2 v2.0.4* (Kim *et al.*, 2019) using default parameters. For each sample, the first aligned base at the 5'end of read 1 was piled up to a CTSS (i.e., Capped-TSS) bed file using custom *Perl* scripts, available at https://github.com/chung-lab/scafe. These CTSS bed files were used for down-sampling, feature intersection and counting.

Processing bulk-ATAC-seq data. The bulk-ATAC-seq data were processed using ENCODE consortium pipelines (https://github.com/kundajelab/atac_dnase_pipelines). The –log(P) signal tracks for pooled replicates were used to define gold-standards for training of the TSS classifiers.

Genome version and gene models. Human genome assembly version hg19 and gene models from GENCODE (Frankish *et al.*, 2019) version v32lift37 were used in all analyses of this study, unless otherwise stated.

References

Buenrostro,J.D. *et al.* (2015) Single-cell chromatin accessibility reveals principles of regulatory variation. *Nature*, **523**, 486–490.

Cumbie,J.S. *et al.* (2015) NanoCAGE-XL and CapFilter: an approach to genome wide identification of high confidence transcription start sites. *BMC Genomics*, **16**, 597.

Fort,A. *et al.* (2014) Deep transcriptome profiling of mammalian stem cells supports a regulatory role for retrotransposons in pluripotency maintenance. *Nat. Genet.*, **46**, 558–566.

Frankish,A. *et al.* (2019) GENCODE reference annotation for the human and mouse genomes. *Nucleic Acids Res.*, **47**, D766–D773.

Frith,M.C. *et al.* (2008) A code for transcription initiation in mammalian genomes. *Genome Res.*, **18**, 1–12.

Heinz,S. *et al.* (2010) Simple combinations of lineage-determining transcription factors prime cis-regulatory elements required for macrophage and B cell identities. *Mol. Cell*, **38**, 576–589.

Kawaji,H. *et al.* (2014) Comparison of CAGE and RNA-seq transcriptome profiling using clonally amplified and single-molecule next-generation sequencing. *Genome Res.*, **24**, 708–717.

Kim,D. *et al.* (2019) Graph-based genome alignment and genotyping with HISAT2 and HISAT-genotype. *Nat. Biotechnol.*, **37**, 907–915.
Kuhn,M. (2008) Building Predictive Models in R Using the caret Package. *J. Stat. Softw.*, **28**, 1–26.
Murata,M. *et al.* (2014) Detecting expressed genes using CAGE. *Methods Mol. Biol. Clifton NJ*, **1164**, 67–85.
Quinlan,A.R. and Hall,I.M. (2010) BEDTools: a flexible suite of utilities for comparing genomic features. *Bioinforma. Oxf. Engl.*, **26**, 841–842.
Siepel,A. *et al.* (2005) Evolutionarily conserved elements in vertebrate, insect, worm, and yeast genomes. *Genome Res.*, **15**, 1034–1050.
Tang,D.T.P. *et al.* (2013) Suppression of artifacts and barcode bias in high-throughput transcriptome analyses utilizing template switching. *Nucleic Acids Res.*, **41**, e44.
Whyte,W.A. *et al.* (2013) Master Transcription Factors and Mediator Establish Super-Enhancers at Key Cell Identity Genes. *Cell*, **153**, 307–319.
